# Public interest in the Brazilian health professions regulation

**DOI:** 10.1590/1518-8345.0000.3114

**Published:** 2019-04-29

**Authors:** Fernando Mussa Abujamra Aith

**Affiliations:** 1 Universidade de São Paulo , Faculdade de Saúde Pública , São Paulo , SP , Brasil .


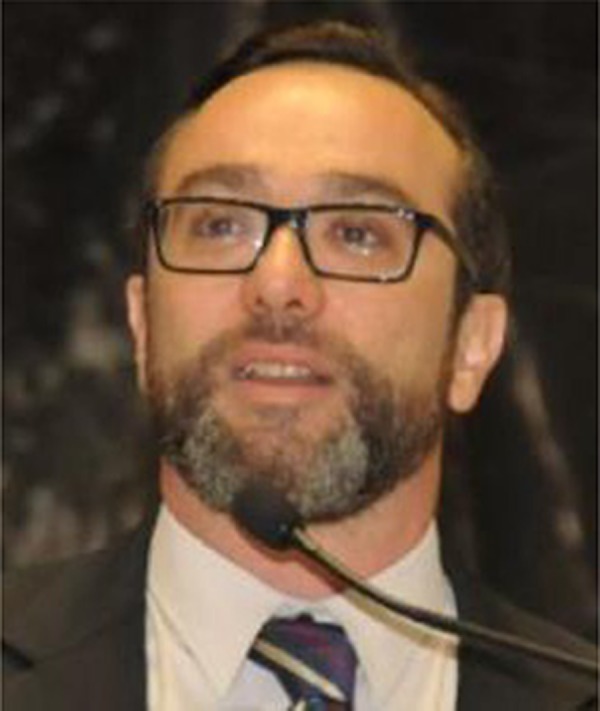


Professional activities related to the provision of health services have always been intensely regulated in modern societies. Now, they are an important area for state action, where quality, efficacy and safety should be ensured, and public interest should be protected against economic, corporate and segmented interests that influence the professional activities carried out in the field of health.

The concept of regulation is polysemic, especially when it is applied to the field of public health^(^ ^1^ ^)^. This text emphasizes the state regulation of health professions, established by the different state institutions responsible for fulfilling the various duties of the Democratic State of Law.

The state regulation of health professions comprises at least three major aspects: i) regulation of the training of professionals who will work in the area of health (college courses and specialization); ii) regulation of professional practice in health (registration, ethics, legal competencies, scope of practice); and iii) regulation of labor relations in the health area (working hours, salaries, career plans).

Thirty years after the creation of the Unified Health System (SUS) in the Federal Constitution, it is imperative to improve the regulatory framework of health professions in Brazil, so that it can harmonize the different interests involved, always defending public interest against corporate or private interests. As established in the Constitution, public interest in the regulation of health professions will always be in line with the construction of a universal, equitable and comprehensive public health system^(^ ^2^ ^)^.

Nowadays, Brazil recognizes 14 health professions that require training in university courses^(^ ^3^ ^)^. These professions have professional councils that have power of self-regulation (since they are formed only by the respective professionals) and state power of regulation (because they are federal autarchies created by law, with their own regulatory and supervisory powers). Thus, in the Brazilian model, the Professional Councils have a hybrid legal nature, since they are at the same time professional self-regulation institutions and state regulatory institutions.

The regulatory framework of health professions in Brazil is characterized by multiple state regulatory institutions, created by various laws. Only in the federal executive branch, the Ministries of Labor, Education, Health and Planning, in addition to thirteen different autonomous Professional Councils, with their own regulatory and supervisory powers, currently have regulatory power over health professions in Brazil. Each of these institutions has the power to define its own rules of regulation in its fields of action, generating a complex set of legal norms that frequently collide with each other.

The creation of the SUS and the expansion of the health sector in Brazil, with its undeniable economic repercussions, has created an expressive amount of regulatory conflicts between the health professions and between the health professions and the state organs responsible for the execution of public policies of SUS. These conflicts cover several topics, such as: the definition of the scope of practice of each profession; the definition of training requirements for the exercise of certain activities; the working hours and salaries of the different professionals.

Considering that the Professional Health Councils sustain their autonomy and are at the same hierarchical level in Public Administration, and that there is no higher administrative body in the Executive Branch that would be able to solve eventual regulatory conflicts, the conflicts between the different institutions have been systematically taken to the Judicial Branch.

In the field of Nursing, two current examples of judicialization of regulatory conflicts clearly demonstrate the problem. The first one refers to Advanced Nursing Practices, established by Resolution no. 568/2018 of the Federal Nursing Council, which allows nurses to work in Nursing Offices and Clinics^(^ ^4^ ^)^. The second example refers to Ordinance MS 2488/2011, which allows nurses to perform nursing consultations, procedures, group activities and, according to protocols and other technical norms established by the federal, state or municipal government agencies or by the Federal district and respecting the legal regulations of the profession, request complementary tests, prescribe medications and refer users to other services, if necessary^(^ ^5^ ^)^. Both processes are currently pending; however, regardless of the judicial response to be given, the mere fact that these issues are being debated in the Judicial Branch and not in the Legislative or in the Executive Branches demonstrates a malfunction of the model that needs to be faced.

It is necessary to create or improve democratic institutions to discuss and resolve possible conflicts in the regulation of health professions in Brazil, balancing public interest, economic and corporate interests, ensuring that public interest always prevail.
